# Overview of Novel Mechanisms in Obesity Pharmacotherapy and Implications for Cardiovascular Disease: A Narrative Review

**DOI:** 10.1007/s11883-026-01438-8

**Published:** 2026-06-26

**Authors:** Michael Bonafede, Aditi Rao, Beverly G. Tchang

**Affiliations:** Comprehensive Weight Control Center, Division of Endocrinology, Diabetes and Metabolism, Weill Cornell Medicine, New York, NY 10021 USA

**Keywords:** Non-GLP-1, GLP-1, Obesity medication, CB1 receptor, Myostatin inhibitor, Selective androgen receptor modulators

## Abstract

**Purpose of review:**

While nutrient-stimulated hormone (NuSH) therapies (e.g., glucagon-like pepide-1 receptor agonists and dual/triple agonists) have transformed the landscape of obesity pharmacotherapy, the next generation of medications may target body composition optimization or other cardiovascular benefits. This review examines novel obesity mechanisms outside of the NuSH class.

**Recent findings:**

Unique mechanisms for obesity treatment include peripherally restricted cannabinoid-1 receptor antagonism, myostatin/activin inhibitors, selective androgen receptor modulators, melanocortin-4 receptor agonism, mitochondrial modulation, thyroid receptor agonists, and fibroblast growth factor analogues. By targeting fat distribution, muscle preservation, inflammatory/oxidative stress pathways, lipid metabolism, and energy expenditure, these agents may improve both the magnitude and quality of weight loss. Early evidence suggests complementary roles alongside NuSH-based therapies for induction, augmentation, and maintenance strategies.

**Summary:**

Several non-NuSH agents have demonstrated potential in preclinical and early clinical studies to optimize body composition, but additional studies are required to prove large-scale, long-term safety and efficacy.

## Introduction

Obesity contributes to atherogenesis through insulin resistance, dyslipidemia, hypertension, endothelial dysfunction, and low-grade inflammation [1, 2]. A landmark primary prevention ASCVD trial found intensive lifestyle intervention for obesity to be ineffective for lowering the risk of incident ASCVD in adults with type 2 diabetes (T2D) [3]. However, a post hoc analysis demonstrated that ASCVD risk was significantly reduced when ≥ 10% weight loss was achieved within the first year, suggesting that the magnitude of weight loss may be critical for ASCVD prevention [4]. Moreover, the quality of weight loss—defined as preferential reduction in fat mass with preservation of lean mass, along with reductions in visceral and hepatic fat, has direct implications for long-term metabolic health and ASCVD risk [2]. Second-generation obesity pharmacotherapies, led by semaglutide, have demonstrated significant protection against ASCVD irrespective of weight loss [5, 6]. Beyond incretin pathways, several novel mechanisms are under active investigation. This review provides a comprehensive overview of these agents by synthesizing mechanistic rationale, efficacy, limitations, and their potential to complement existing therapies in clinical practice.

## Methods

A comprehensive literature search was conducted using PubMed to identify relevant articles focusing on novel pharmacotherapy for obesity, supported by expert opinions and artificial intelligence (AI)-based tools, OpenAI *ChatGPT* (GPT-5.1) and OpenEvidence. The search employed the following Boolean search strategy, with restriction to the English language: (“weight loss” OR “obesity”) NOT (“GLP-1” OR “surgery” OR “diet” OR “lifestyle” OR “exercise” OR “physical activity”) AND (“pharmacotherapy” OR “medications” OR “medication”). These AI tools were used primarily for additional literature identification. For ease of reference, emerging pharmacotherapies have been consolidated in Table 1 according to mechanism of action, along with their corresponding weight loss efficacy and key preclinical and clinical data.

**Table 1:** Emerging obesity medications by mechanism

Agent	Mechanism	Preclinical data	Clinical data	Weight-loss range (key trials)	Duration of trial(s)	Notes/potential
**CRB-913**	CB1 inverse agonist	DIO mice: ~22–38% WL; ↓ hepatic steatosis; synergy w/GLP-1/GIP agonists [7, 8]	Phase 1 SAD/MAD in healthy volunteers: early safety/tolerability; no efficacy readout yet [9]	Preclinical: ~22–38% WL; Human pending	Preclinical: 18–30 days; Phase 1: 2–4 weeks	Peripherally restricted
**Nimacimab**	CB1 mAb negative allosteric modulator	~ 20–25% WL mono; >30% w/tirzepatide; blunts rebound [10]	Phase 2a obesity: nimacimab+semaglutide ≈ 13% WL vs. ≈ 10% semaglutide alone [11]	Preclinical: up to ~ 30%; Human: ~0–13% depending on dose/combination	Preclinical: 8–12 wks; Phase 2: ~26 wks	Peripherally restricted.
**Bimagrumab**	ActRII (myostatin/activin) antagonist mAb	↑ lean mass; ↓ fat mass; ↑ FAO; improved insulin sensitivity [12, 13]	48-wk RCT: −20% fat mass; +3–4% lean mass; modest BW loss (~ 3–6%) [14]	~ 3–6% WL clinically, but large shift toward fat mass loss	48 weeks	Improves composition more than total weight loss
**Trevogrumab**	ActRII-targeting antibody/ligand trap	Improved body composition w/GLP-1 RA in models [12, 13]	Phase 2 interim: greater fat loss + lean preservation [15]	Mid-single to low-double digit % WL (combo-dependent)	~ 26 weeks	Used with semaglutide ± garetosmab in triplet regimens
**Enobosarm**	SARM	Anabolic + anti-catabolic muscle effects [16]	Phase 2b w/sema: −1.2% vs. − 4.1% lean loss; ↑ proportion fat loss [17]	Minimal incremental WL (total ~ 10–12% driven by sema)	16 weeks + extension	Optimizes lean mass preservation, especially older adults
**Bremelanotide**	MC4R agonist	↓ appetite; ↑ EE; BAT activation in models [18]	~ 1–1.5 kg over 2 wks (mono); ~4–5% in combo [19, 20]	Human: ~1–5%	2–8 weeks	Adjunct to incretins; may increase blood pressure and heart rate
**Vutiglabridin**	Mitochondrial/inflammation modulator	~ 20–25% WL rodents; ↓ steatosis; ↑ PON1 [21–23]	Phase 1: no safety concerns; no significant WL [24, 25]	Preclinical: ~20–25%; Human: none meaningful	6–12 wks preclinical; 14 days human	Targets hepatic steatosis + inflammation
**Pegozafermin**	FGF analogue	Improves liver steatosis/fibrosis + lipids [26]	Phase 2 NASH: modest WL (~ 3–6%) [27, 28]	~ 3–6% WL clinically	12–24 weeks	Primarily hepatic benefit
**Resmetirom (MGL-3196)**	Selective TRβ agonist	↑ hepatic FAO; ↓ liver fat; improved LDL-C/apoB [29]	Phase 3 MASH: no meaningful WL vs. placebo [30]	No clinically significant WL	52 + weeks	Liver + lipid targeted agent

ActRII, activin receptor II; apoB, apolipoprotein B; BAT, brown adipose tissue; CB1, cannabinoid receptor-1; DIO, diet-induced obese; EE, energy expenditure; FAO, fatty acid oxidation; FGF, fibroblast growth father; GLP-1, glucagon-like peptide-1; GLP-1 RA, GLP-1 receptor agonist; GIP, glucose-dependent insulinotropic peptide; LDL-C, low-density lipoprotein-cholesterol; mAb, monoclonal antibody; MAD, Multiple Ascending Dose; MASH, metabolic dysfunction-associated steatohepatitis; MC4R, melanocortin-4 receptor; PON1, paraoxonase 1; RCT, randomized controlled trial; SAD, Single Ascending Dose; SARM, selective androgen receptor modulator; TRβ, thyroid hormone receptor-beta; WL, weight loss

## Endocannabinoid System Modulators: Peripherally Restricted CB1 Receptor Antagonists and Inverse Agonists

### Background: Rationale and Mechanism of Action

The endocannabinoid system regulates appetite, energy expenditure, lipogenesis, and insulin sensitivity [9]. CB1 receptors are expressed centrally and peripherally, including in the liver, adipose tissue, skeletal muscle, and pancreas. Activation of CB1 receptors increases appetite, enhances de novo lipogenesis, and impairs insulin signaling through downstream effects on adenosine monophosphate–activated protein kinase (AMPK) and sterol regulatory element-binding protein 1c (SREBP-1c) pathways. Conversely, CB1 blockade reverses these processes, enhancing mitochondrial fatty acid oxidation, improving hepatic insulin sensitivity, and reducing hepatic and visceral fat stores [9].

Inhibition of CB1 receptors has historically demonstrated weight loss. Centrally penetrating CB1 antagonists, such as rimonabant, promoted weight loss and improved triglyceride and high-density lipoprotein cholesterol levels but were withdrawn due to neuropsychiatric adverse effects in 2008 [31]. In pooled phase 3 trials from the RIO program (*n* ≈ 4,100), rimonabant 20 mg daily was associated with approximately 2.5-fold higher odds of depressed mood disorders and three-fold higher odds of anxiety-related discontinuation compared with placebo, despite exclusion of individuals with active depression [32].

Peripherally restricted CB1 antagonists/inverse agonists aim to retain metabolic benefits (e.g., reduced hepatic steatosis, increased lipolysis, improved insulin sensitivity) while minimizing neuropsychiatric side effects by limiting brain penetration.

### Agents and Evidence

CRB-913 is a next-generation, peripherally restricted oral CB1 inverse agonist. In diet-induced obese (DIO) mice, CRB-913 produced substantial weight and fat-mass reductions and reduced liver triglycerides, a remarkable 22% weight loss in 18 days. Combination with incretin therapies further enhanced weight loss in preclinical models, reaching 32.6% weight loss in combination with tirzepatide at day 18 [7]. Phase Ia data has recently been reported, finding CRB-913 to be safe and well-tolerated across all doses with no signal in neuropsychiatric assessments. Preliminary results revealed a placebo-adjusted mean weight loss of 2.9% at Day 14, however the cohort was small (*n* = 12) and a 12-week dose-finding study in people with obesity is expected to be completed in summer 2026.

Nimacimab is a monoclonal antibody and negative allosteric modulator targeting peripheral CB1 that, due to size, has minimal central nervous system (CNS) penetration. In preclinical humanized CB1 models, nimacimab achieved ~ 20–25% weight loss as monotherapy and > 30% when combined with tirzepatide; it also blunted post-tirzepatide rebound weight gain in DIO models [10]. In the 26-week Phase 2a CBeyond trial, nimacimab 200 mg subcutaneous weekly injection as monotherapy produced a mean weight change of − 1.52% versus − 0.26% for placebo for a placebo-adjusted change of − 1.26% (95% CI − 3.5 to 1.0%) [11]. When combined with semaglutide, weight loss was − 13.2% versus − 10.25% for semaglutide-alone, with a statistically significant between-group difference (− 2.95%, *p* = 0.0372). Crucially, no neuropsychiatric adverse events were observed. These Phase 2a trial results are not yet published, and it is important to note that monotherapy did not meet its primary endpoint, although the potential for combination therapy may be promising.

In a randomized, double-blind pilot trial in adults with obesity, the CB1 receptor modulator nabilone (2 mg/day and 6 mg/day arms) showed a statistically significant treatment effect on body weight and body mass index (BMI) in the low-dose arm versus placebo after 12 weeks, although the study was terminated early due to poor tolerability in the high-dose arm (all four participants withdrew) and thus remains limited in scope [33].

### Implications for Obesity and CVD

There is evidence of robust fat-mass reduction in preclinical models for these agents; early human signals suggest potential for clinically relevant weight loss and metabolic improvements. Peripheral CB1 antagonism may preferentially reduce visceral and hepatic fat, which aligns with improved insulin sensitivity and atherogenic dyslipidemia [8]. Given history of rimonabant, the class must definitively demonstrate long-term psychiatric safety, even with peripheral restriction, and establish gastrointestinal (GI) or hepatic safety in larger trials. By reducing visceral/ectopic fat and inflammation, CB1 antagonism may indirectly reduce ASCVD risk, and combination with NuSH agents may amplify its benefits [7]. Outcome data in cardiovascular populations, however, remain to be established.

## Myostatin/Activin Inhibitors

### Background: Rationale and Mechanism of Action

Myostatin/activin signaling through activin type II receptors (ActRIIA/IIB) negatively regulates skeletal muscle growth and influences adipose biology. Physiologically, binding of myostatin (aka. growth differentiation factor-8 (GDF-8)) and activin to ActRIIA/IIB reduces muscle protein synthesis and increases muscle protein breakdown, with activin A having a higher affinity for ActRIIA/IIB receptors than activin B [12]. Blocking this pathway via ActRII signaling thereby increases muscle mass and may enhance resting energy expenditure, fatty-acid oxidation, and insulin-mediated glucose disposal [13]. This mechanism enhances the quality of weight loss by preserving muscle mass, which may increase functional capacity and improve cardiometabolic health.

### Agents and Evidence

Bimagrumab (BYM338) is a monoclonal antibody that inhibits myostatin/activin signaling by binding to ActRIIA/IIB receptors. In a 48-week RCT in adults with type 2 diabetes and obesity, bimagrumab improved total fat mass by − 20.5%, compared to − 0.5% in the placebo arm. Additionally, statistically significant lean mass preservation was observed, with an increase of 3.6% lean mass, as compared to − 0.8% in the placebo arm [14]. Notably, low-density lipoprotein-cholesterol (LDL), HDL, and triglycerides did not appear to improve in the bimagrumab group compared to placebo, and high-sensitivity CRP (hsCRP) increased in the bimagrumab group while it decreased in the placebo group. Combination therapy with semaglutide has demonstrated a greater proportion of fat mass loss and improved lean-mass preservation compared with semaglutide alone in preliminary phase 2 data presented at the American Diabetes Association 2025 Scientific Sessions [34]. A phase 2 placebo-controlled study is also underway evaluating bimagrumab and tirzepatide, alone or in combination in people with obesity, further solidifying the emerging demand for combination therapy.

Trevogrumab is an anti-myostatin antibody under evaluation in combination with semaglutide. Interim analyses suggest improved fat-mass reduction and lean-mass preservation compared with semaglutide monotherapy [15].

### Implications for Obesity and CVD

This class is distinguished by improvements in body composition: substantial fat-mass loss with preservation or gain of lean mass, rather than dramatic total weight loss alone. Such changes are associated with improved insulin sensitivity, as demonstrated with bimagrumab, while purported improvements in energy expenditure, functional metrics, and sarcopenia risk remain to be proven in clinical trials. The absolute change in total body weight may be modest versus NuSH-based agents; parenteral administration and cost/availability are practical considerations [34]. By preserving muscle and reducing visceral fat, ActRII antagonists may indirectly improve ASCVD risk, but future studies will need to discern its effect on cholesterol and inflammatory markers. Further, off-target effects at cardiomyocytes may limit its benefits in certain diseases such as heart failure with preserved ejection fracture, in which semaglutide has demonstrated benefit potentially via cardiac remodeling [35]. While current evidence does not purport bimagrumab has adverse effects on human cardiac structure or cardiac injury biomarker signal over ~ 6 months of exposure, newer Phase 2 data has shown up to a 17.6% increase in LDL cholesterol with bimagrumab 30 mg/kg, leaving net cardiovascular risk uncertain [36]. 

Definitive cardiovascular outcome data are not yet available.

## Selective Androgen Receptor Modulators

### Background: Rationale and Mechanism of Action

Selective androgen receptor modulators (SARMs) activate the androgen receptor with tissue selectivity to drive anabolic effects in muscle and bone while minimizing androgenic adverse effects [16]. In obesity management, particularly in older adults, preserving or increasing lean mass during weight loss may help maintain resting energy expenditure, functional status, and glucose tolerance [37], potentially supporting long-term weight maintenance. Mechanistically, selective androgen receptor activation in skeletal muscle promotes myofibrillar protein synthesis, satellite-cell recruitment, and downregulation of proteolytic pathways (e.g., MuRF-1, Atrogin-1), thereby preserving muscle during catabolic states [16].

### Agent and Evidence

Enobosarm is a SARM that has been previously studied in patients with cancer or cachexia, where it demonstrated efficacy in increasing lean mass (median increase of 1.0 kg with 3 mg/d, 1.5 kg with 1 mg/d, and 0.02 kg with placebo) alongside improved function [38].

In a randomized Phase II trial of older adults with muscle wasting, enobosarm increased lean body mass and improved functional measures such as stair-climb power, providing a rationale for its role in preventing sarcopenia [37]. Unlike non-selective androgens, enobosarm achieves its effects with minimal activation in reproductive tissues due to tissue-specific co-activator recruitment, allowing anabolic benefit without typical androgenic adverse effects. By maintaining muscle mass and thus resting energy expenditure and glucose disposal, enobosarm may mitigate sarcopenic decline during weight loss and support durable metabolic health.

Enobosarm, when combined with semaglutide in the Phase 2b QUALITY study, reduced lean-mass loss by ~ 70% (− 1.2% vs. − 4.1% with semaglutide alone; *p* = 0.002) and increased proportional fat-mass loss (− 10.9% vs. − 8.6%), supporting its role in improving the quality of weight loss rather than the absolute magnitude [17]. Safety signals to monitor include lipid changes, hepatotoxicity, androgenic effects, and sex-specific considerations [39].

### Implications for Obesity and CVD

Focusing on body-composition quality, i.e., lean mass preservation, rather than large absolute weight loss, SARMs have the potential to avoid sarcopenic decline and thereby preserve metabolic reserve, reduce insulin resistance, and potentially improve vascular outcomes [37]. Monitoring for androgenic effects and lipid changes is critical, as well as tailored population selection (e.g., older adults, sarcopenia risk). Improved muscle mass and function may aid glycemic control and cardiorespiratory fitness, with potential ASCVD benefit over time.

## Melanocortin-4 Receptor Agonists

### Background: Rationale and Mechanism of Action

Melanocortin-4 receptor (MC4R) is a central regulator of appetite and energy expenditure. Pharmacologic activation reduces food intake and can increase energy expenditure, including brown adipose tissue thermogenesis. Because MC4R activation may increase sympathetic tone, heart rate, and blood pressure, cardiovascular safety must be carefully evaluated in obesity populations [18]. Currently, only one MC4R activator is FDA-approved, setmelanotide, which is a once-daily injection approved for the treatment of specific genetic obesity diseases: pro-opiomelanocortin (POMC) deficiency, proprotein subtilisin/kexin type 1 (PCSK1) deficiency, leptin receptor (LEPR) deficiency, or Bardet-Biedl syndrome.

### Agent and Evidence

Bremelanotide is a melanocortin receptor agonist currently approved for hypoactive sexual desire disorder (HSDD) in premenopausal women. While clinical trials of bremelanotide in HSDD did not observe weight loss as a side effect, phase 1 data in premenopausal women with obesity suggested some efficacy. Two short-term randomized, double-blind Phase 1 trials in pre-menopausal women with obesity reported early proof-of-concept for weight reduction and appetite–intake suppression (referred to as Study A and B).

In Study A, after 16 days of thrice-daily subcutaneous dosing, the mean difference versus placebo in body weight was 1.3 kg (95% CI − 1.9 to − 0.8; *p* < 0.0001); however, mean caloric intake among bremelanotide-treated subjects decreased by about 400 kcal/day versus placebo (*p* < 0.01). In Study B (crossover, 4-day periods), twice-daily bremelanotide produced mean weight loss of 1.7 kg versus 0.9 kg with placebo (*p* < 0.001) and caloric intake reductions of 398–469 kcal/day (*p* < 0.0001). Adverse events were mostly mild; injection-site reactions, nausea, dizziness, transient blood-pressure elevation and skin-pigmentation changes (reported in 15–63% across both studies) occurred, and five participants withdrew due to nausea, dizziness or hypertension [19]. These data indicate that MC4R agonism via bremelanotide can rapidly reduce caloric intake and induce short-term weight loss in obesity, though the effect size is modest and longer-term trials are needed to determine durability and safety.

Subsequently, a company press release shared partial phase 2 data illustrating modest weight loss when combined with tirzepatide [20]. Mean body weight reduction was 4.4% with bremelanotide (1.25 mg/d) plus tirzepatide (2.5 mg/wk) vs. 1.6% with placebo over 8 weeks. Published obesity-specific clinical efficacy remains limited; future formulations or molecules that engage MC4R with greater selectivity or biased signaling may improve tolerability and efficacy.

### Implications for Obesity and CVD

These agents are mechanistically promising for appetite suppression and potential energy-expenditure increases, although definitive obesity outcomes require longer and more robust clinical trials. CNS-mediated increases in heart rate or blood pressure may limit use in patients with ASCVD or uncontrolled hypertension [40]. Regarding CVD outcomes, while weight loss could reduce risk, hemodynamic effects might offset benefit in some patients; careful selection and monitoring will be necessary depending on drug safety profiles.

## Mitochondrial Modulators

### Background: Rationale and Mechanism of Action

Vutiglabridin is a synthetic analogue of glabridin. Glabridin is a licorice-derived isoflavan that activates AMPK in liver and skeletal muscle, leading to suppressed lipogenesis and enhanced fatty-acid β-oxidation. It is also thought to improve mitochondrial function, referring to AMPK-driven increases in mitochondrial fatty-acid oxidation and respiratory capacity, promotion of mitochondrial biogenesis via peroxisome proliferator-activated receptor gamma coactivator 1-alpha (PGC-1α) dependent programs, thereby reducing oxidative stress and lipid accumulation within hepatocytes and myocytes [41].

### Agent and Evidence

Vutiglabridin: In obese rodent models, vutiglabridin produced meaningful weight reduction, reduced hepatic fat, and improved inflammatory and oxidative stress markers [21, 22]. In DIO mice, 6-week oral treatment with the glabridin analogue vutiglabridin at 10, 30, and 100 mg/kg reduced body weight by 8.3%, 21%, and 40%, respectively, compared with controls [23]. Phase 1 single- and multiple-ascending-dose studies in healthy men demonstrated acceptable safety profiles and favorable biomarker shifts to favor anti-inflammation (e.g., increase in anti-inflammatory adiponectin, decrease in pro-inflammatory C-C motif chemokine ligand 2 (CCL2)) [24], supporting further Phase II obesity efficacy trials to define dose–response and long-term safety.

### Implications for Obesity and CVD

Early-stage data suggest potential for weight loss and improvements in hepatic and inflammatory biomarkers relevant to metabolic syndrome. Efficacy and long-term safety data in humans are lacking [25]. If the observed phase 1 changes in inflammatory profiles translate to phase 2 and phase 3 studies, ASCVD risk could be favorably impacted.

## Thyroid Hormone Receptor Agonist

### Mechanism of Action and Rationale

Thyroid hormone receptor beta (TRβ) is expressed in liver and adipose tissue and is involved in energy and lipid metabolism. Selective agonists mediate energy expenditure, promote thermogenesis, and improve glucose and lipid metabolism [29].

Resmetirom (MGL-3196) is a selective TRβ agonist and is the first and only TRβ agonist approved for the treatment of noncirrhotic metabolic dysfunction-associated steatohepatitis (MASH) with moderate to advanced fibrosis. Phase 3 trial data at 80 mg and 100 mg of resmetirom showed improvements in liver stiffness (− 1.02, − 1.70), hepatic fat (− 28.8, − 33.9), LDL cholesterol (− 11.1%, − 12.6%), apolipoprotein B (− 15.6%, − 18.0%), and triglycerides (− 15.4%, − 20.4%), as well as acceptable safety and tolerability [30]. However, there were no significant differences in weight compared with placebo.

### Agent and Evidence

TERN-501 is another TRβ agonist which showed promising data in phase 2a trials as monotherapy or when combined with TERN-101, a farnesoid X receptor agonist. TERN-501 showed improvements in liver fat content − 15.4% with 1 mg, − 27.5% with 3 mg and − 44.8% with 6 mg. Treatment with TERN-501 also led to dose-dependent changes in lipids including decrease from baseline in apolipoprotein B, total cholesterol, triglycerides, VLDL (very low-density lipoprotein) and lipoprotein(a). However, there were no changes in weight seen across treatment groups [42].

CG-0416 is a novel TRβ agonist with specific liver targeting currently being investigated for both MASH and obesity in the preclinical phase. One preclinical study aimed to evaluate efficacy in treating obesity in DIO mice as monotherapy and in combination with GLP-1RAs. After 28 days, monotherapy led to weight losses of 9.6% at the 1 mg/kg dose, 15.4% at the 3.3 mg/kg and 21.6% at the 10 mg/kg dose. Further, about 95% of the weight loss was fat mass loss. Combination with semaglutide led to faster and greater weight loss (40.1% vs. 24%) at 28 days. Overall treatment with CG-0416 led to improved lipid and glucose control with an acceptable safety profile.

### Implications for Obesity and CVD

Although prior thyroid hormone receptor beta agonists have shown significant improvements in liver histology and cholesterol levels, none until now have shown promising findings in weight loss. While the precise mechanism for why CG-0416 demonstrates meaningful weight reduction in contrast to resmetirom and TERN-501 is not fully elucidated, it may be due to its design as a liver-target prodrug, causing enhanced intrahepatic exposure and potentially amplifying systemic energy expenditure. CG-0416 is a novel therapy that can lead to significant improvements in fat loss while preserving lean mass and improving lipid and glucose metabolism, mirroring crucial goals in the treatment of obesity and ASCVD. Further human clinical data is needed to establish long-term safety and efficacy.

## Fibroblast Growth Factor Analogue

### Mechanism of Action and Rationale

Fibroblast growth factor 21 (FGF21) is a peptide hormone secreted by the liver that regulates lipid and glucose metabolism and energy homeostasis. Pegozafermin is a long-acting glycopegylated recombinant FGF21 analogue [27]. It is currently being developed for the treatment of MASH and severe hypertriglyceridemia.

### Agent and Evidence

Administration of pegozafermin to diabetic, obese monkeys showed improvement in lipid levels, glucose levels, liver enzymes, food intake and body weight [26]. A Phase 1B/2A study showed acceptable safety and tolerability as well as improvements in hepatic steatosis, inflammatory markers, lipid and glucose levels. The study also demonstrated a statistically significant decrease in body weight of 2.18% at week 12 compared to placebo [27]. Effects on food intake and body weight are not conclusive with differing results, as a phase 2B trial showed treatment with pegozafermin led to improvements in hepatic fibrosis but no change in weight [28]. Ongoing phase 3 trial for patients with hypertriglyceridemia are underway. Most side effects demonstrated were mild-moderate GI symptoms.

### Implications for Obesity and CVD

Although pegozafermin has not demonstrated meaningful weight loss and is primarily being pursued for MASH and hypertriglyceridemia, its role as an FGF21 analog modulates energy homeostasis, including lipid metabolism, insulin sensitivity, and energy expenditure. These benefits may complement weight-centric therapies in the future, especially if future formulations demonstrate meaningful weight loss. No studies exist showing efficacy in treating obesity yet, however its benefits in treating metabolic conditions including hepatic steatosis, hepatic fibrosis, elevated triglycerides and others can lead to improved CVD outcomes.

## Discussion

The emerging pharmacotherapies collectively reviewed in this paper reflect a shift from appetite-centric approaches toward targeting the underlying biology of obesity, including energy expenditure, adipose tissue distribution, muscle preservation, and metabolic regulation. Despite heterogeneity in mechanism, these agents share the goal of improving both magnitude and quality of weight loss, with potential downstream benefits on cardiometabolic health and ASCVD risk.

Careful attention to safety and patient selection must be noted for all classes. For example, CB1 antagonists must demonstrate durable psychiatric safety; MC4R agonists require hemodynamic monitoring; SARMs and ActRII antagonists require vigilance for lipid effects; thyroid receptor agonists require cardiac monitoring; and fibroblast growth factor analogues require gastrointestinal monitoring.

Combination with NuSH-based therapies is a prominent development theme: CB1 antagonists and ActRII antagonists may augment fat-mass loss while preserving lean mass; SARMs may mitigate lean-mass loss during aggressive weight reduction; and thyroid receptor agonists may allow for greater weight loss, improved lipid metabolism with more muscle preservation. Such plural-mechanism approaches could optimize both magnitude and quality of weight loss and improve durability, all of which are key goals in obesity management with downstream ASCVD implications.

Limitations of this investigation are intrinsic to its study design as a narrative review, which used a single database (e.g., PubMed) instead of multiple complementary databases for its literature search and relied on professional expert opinion for additional guidance, which may introduce bias. The specific agents highlighted in this review were selected to emphasize less extensively characterized mechanistic pathways, as these targets have received less focus in the literature compared with amylin, GIP, GLP-1, and glucagon-based therapies. Several novel agents were outside the scope of this review, such as gene therapy using CRISPR-based technology. Further, several outcomes reported relied on industry press releases and/or poster presentations, given the lack of peer-reviewed publications.

While there are many novel therapeutics being developed for the treatment of obesity and established ASCVD, there are important limitations to acknowledge. Firstly, there is limited long-term efficacy and safety data. Long-term human outcomes, including sustained weight loss, CV risk reduction, and adverse event profiles, remain largely unknown for these therapies. Furthermore, emerging therapies may be expensive, and access can be limited by regulatory approval status, insurance coverage, and geographic disparities.

## Conclusions

Mechanistically novel obesity agents, including peripheral CB1 antagonists, ActRII antagonists, SARMs, MC4R agonists, mitochondrial modulators (e.g., vutiglabridin), thyroid receptor agonists, fibroblast growth factor analogues, and more, all have the potential to expand the therapeutic toolkit beyond anorexigenic pathways. By targeting fat distribution, ectopic fat, muscle preservation, inflammatory/oxidative stress pathways, lipid metabolism, and energy expenditure, these agents may improve both the magnitude and quality of weight loss. Early evidence suggests complementary roles alongside NuSH-based therapies for induction, augmentation, and maintenance strategies. Robust, adequately powered clinical trials are needed to quantify obesity efficacy in humans, long-term safety, and cardiometabolic outcomes, particularly in patients with established ASCVD.

## Key References

- Shahid I, Zakaria F, Chang R, et al. Obesity and atherosclerotic cardiovascular disease: a review of social and biobehavioral pathways. *Methodist DeBakey Cardiovasc J*. 2025;21:23–34. 10.14797/mdcvj.1528.
  - ⚬ Comprehensive review detailing the social, behavioral, and biological pathways linking obesity to atherosclerotic cardiovascular disease risk.
- Crater G, et al. Effects of the CB1 receptor antagonist INV-202 in metabolic syndrome: a randomized placebo-controlled phase 1b study. *Diabetes Obes Metab.* 2024;26:1090–1097. 10.1111/dom.15488.
  - ⚬ Early-phase clinical study demonstrating metabolic effects and short-term safety of peripherally restricted CB1 antagonism in humans, supporting further investigation of this pathway for obesity and cardiometabolic disease.

## Data Availability

All data supporting the findings of this study are available within the paper and cited appropriately in the reference list.

## References

[1] Shahid I, Zakaria F, Chang R, et al. Obesity and atherosclerotic cardiovascular disease: a review of social and biobehavioral pathways. Methodist DeBakey Cardiovasc J. 2025;21:23–34. 10.14797/mdcvj.1528. 39990759 10.14797/mdcvj.1528 PMC11843985

[2] Bays HE, Kirkpatrick CF, Maki KC, et al. Obesity, dyslipidemia, and cardiovascular disease: a joint expert review from the Obesity Medicine Association and the National Lipid Association 2024. J Clin Lipidol. 2024;18:e320–50. 10.1016/j.jacl.2024.04.001. 38664184 10.1016/j.jacl.2024.04.001

[3] Look AHEADR, Group, Wing RR, Bolin P, et al. Cardiovascular effects of intensive lifestyle intervention in type 2 diabetes. N Engl J Med. 2013;369:145–54. 10.1056/NEJMoa1212914. 23796131 10.1056/NEJMoa1212914 PMC3791615

[4] Look AHEADR, Group, Gregg EW, Jakicic JM, et al. Association of weight loss and physical fitness with long-term cardiovascular outcomes in type 2 diabetes: a post hoc analysis of the Look AHEAD trial. Lancet Diabetes Endocrinol. 2016;4:913–21. 10.1016/S2213-8587(16)30162-0. 27595918 10.1016/S2213-8587(16)30162-0 PMC5094846

[5] Lincoff AM, Brown-Frandsen K, Colhoun HM, et al. Semaglutide and cardiovascular outcomes in obesity without diabetes. N Engl J Med. 2023;389:2221–32. 10.1056/NEJMoa2307563. 37952131 10.1056/NEJMoa2307563

[6] Deanfield J, Verma S, Scirica BM, et al. Semaglutide and cardiovascular outcomes in patients with obesity and prevalent heart failure: a prespecified analysis of the SELECT trial. Lancet. 2024;404:773–86. 10.1016/S0140-6736(24)01498-3. 39181597 10.1016/S0140-6736(24)01498-3

[7] Morningstar M, Kolodziej A, Ferreira S, et al. Novel cannabinoid receptor 1 inverse agonist CRB‐913 enhances efficacy of tirzepatide, semaglutide, and liraglutidein the diet‐induced obesity mouse model. Obesity Silver Spring. 2023;31:2676–88. 10.1002/oby.23902. 37840407 10.1002/oby.23902

[8] Tam J, Vemuri VK, Liu J, et al. Peripheral CB1 cannabinoid receptor blockade improves cardiometabolic risk in mouse models of obesity. J Clin Invest. 2010;120:2953–66. 10.1172/JCI42551. 20664173 10.1172/JCI42551 PMC2912197

[9] Crater G, et al. County‐level socio‐environmental factors and obesity prevalence in the United States. Diabetes Obes Metab. 2024;26:1090–7. 10.1111/dom.15488. 38356053 10.1111/dom.15488 PMC11447680

[10] Ruiz-Pino F, Muñoz E, Ponce-Díaz FJ, et al. 1716-P: Nimacimab, a peripherally restricted CB1 inhibitor, promotes metabolic homeostasis in a diet-induced obesity (DIO) mouse model as demonstrated by weight loss, restored hormonal regulation, and reduced inflammatory biomarkers. Diabetes. 2025;74(Suppl 1):1716-P. 10.2337/db25-1716-P.

[11] Skye Bioscience Inc. Top-line CBeyond™ phase 2a data for nimacimab in obesity. In: Skye Bioscience. 2025. https://www.skyebioscience.com. Accessed 1 Nov 2025.

[12] Lach-Trifilieff E, et al. Gut microbiota metabolism of dietary fiber influences allergic airway disease and hematopoiesis. Nat Med. 2014;126. 10.1038/nm.3444. 10.1038/nm.3444 24390308

[13] Han HQ, Zhou X, Mitch WE, Goldberg AL. Myostatin/activin pathway antagonism: molecular basis and therapeutic potential. Int J Biochem Cell Biol. 2013;45:2333–47. 10.1016/j.biocel.2013.05.019. 23721881 10.1016/j.biocel.2013.05.019

[14] Heymsfield SB, et al. Effect of Bimagrumab vs placebo on body fat mass among adults with type 2 diabetes and obesity. JAMA Netw Open. 2021;4:e2033457. 10.1001/jamanetworkopen.2020.33457. 33439265 10.1001/jamanetworkopen.2020.33457 PMC7807292

[15] Regeneron Pharmaceuticals. Interim results from the phase 2 COURAGE trial. In: Regeneron. 2025. https://www.regeneron.com. Accessed 1 Nov 2025.

[16] Dalton JT, et al. Selective androgen receptor modulators in clinical trials. Endocr Rev. 2021;42:240–56. 10.1210/endrev/bnaa037.

[17] Halsey G. Enobosarm preserves lean mass with semaglutide: phase 2b data. In: Patient Care Online. 2025. https://www.patientcareonline.com. Accessed 1 Nov 2025.

[18] Tao YX. The melanocortin-4 receptor pathway in obesity. Endocr Rev. 2010;31:506–43. 10.1210/er.2009-0037. 20190196 10.1210/er.2009-0037 PMC3365848

[19] Spana C, Jordan R, Fischkoff S. Persistence with insulin pump therapy among children and young adults with type 1 diabetes in Germany: an update. Diabetes Obes Metab. 2022;24:1084–93. 10.1111/dom.14647. 35014155 10.1111/dom.14647

[20] Palatin Technologies Inc. Bremelanotide with tirzepatide meets phase 2 endpoint. In: Palatin Technologies. 2025. https://www.palatint.com. Accessed 1 Nov 2025.

[21] Sulaiman D, et al. Vutiglabridin modulates paraoxonase 1 and ameliorates diet-induced obesity in hyperlipidemic mice. Biomolecules. 2023;13:687. 10.3390/biom13040687. 37189434 10.3390/biom13040687 PMC10135725

[22] Choi LS, Jo IG, Kang KS, et al. Discovery and preclinical efficacy of HSG4112, a synthetic structural analog of glabridin, for the treatment of obesity. Int J Obes. 2021;45(1):130–42. 10.1038/s41366-020-00686-1. 10.1038/s41366-020-00686-1 PMC7752758 32943760

[23] Lee HM, Lee JH, Kim SH, et al. Vutiglabridin ameliorates obesity by directly reducing fat mass through AMPK/lipophagy activation in adipocytes. Biomed Pharmacother. 2025;193:118759. 10.1016/j.biopha.2025.118759. 41237461 10.1016/j.biopha.2025.118759

[24] Na JY, et al. Non‐synonymous alterations in AKR7A3 and ABCA6 correlate with bleeding in aged patients treated with rivaroxaban. Clin Transl Sci. 2022;1185. 10.1111/cts.13205. 10.1111/cts.13205 PMC9010266 34859601

[25] Na JY, Yoon DY, Yoo H, et al. Safety, tolerability, pharmacokinetic, and pharmacodynamic characteristics of vutiglabridin: a first‐in‐class, first‐in‐human study. Clin Transl Sci. 2022;15:2744–57. 10.1111/cts.13401. 36176051 10.1111/cts.13401 PMC9652434

[26] Rosenstock M, et al. GlycoPEGylated FGF21 analog pegozafermin. J Pharmacol Exp Ther. 2023;387:204–13. 10.1124/jpet.123.001741. 37562970 10.1124/jpet.123.001618

[27] Loomba R, et al. Pegozafermin in non-alcoholic steatohepatitis. Lancet Gastroenterol Hepatol. 2023;8:120–32. 10.1016/S2468-1253(22)00360-1. 36521501 10.1016/S2468-1253(22)00347-8

[28] Loomba R, et al. Randomized, controlled trial of the FGF21 analogue pegozafermin in NASH. N Engl J Med. 2023;389:998–1008. 10.1056/NEJMoa2304286. 37356033 10.1056/NEJMoa2304286 PMC10718287

[29] Castillo M, et al. Impaired metabolic effects of a thyroid hormone receptor beta-selective agonist in a mouse model of diet-induced obesity. Thyroid. 2010;20:545–53. 10.1089/thy.2009.0318. 20406106 10.1089/thy.2009.0318 PMC2941403

[30] Harrison SA, et al. A phase 3, randomized, controlled trial of resmetirom in NASH with liver fibrosis. N Engl J Med. 2024;390:497–509. 10.1056/NEJMoa2309000. 38324483 10.1056/NEJMoa2309000

[31] Christensen R, Kristensen PK, Bartels EM, Bliddal H, Astrup A. Efficacy and safety of the weight-loss drug rimonabant: a meta-analysis of randomised trials. Lancet. 2007;370:1706–13. 10.1016/S0140-6736(07)61721-8. 18022033 10.1016/S0140-6736(07)61721-8

[32] Van Gaal LF, Rissanen AM, Scheen AJ, Ziegler O, Rössner S, RIO-Europe Study Group. Effects of rimonabant on weight reduction and cardiovascular risk factors: the RIO-Europe study. Lancet. 2005;365:1389–97. 10.1016/S0140-6736(05)66374-X. 15836887 10.1016/S0140-6736(05)66374-X

[33] Matheson J, Tertigas D, Malik S, et al. Feasibility and tolerability of nabilone for the treatment of obesity: a randomized controlled pilot trial. Cannabis Cannabinoid Res. 2025;10:640–51. 10.1089/can.2025.0034. 40353878 10.1089/can.2025.0034

[34] Regeneron Pharmaceuticals. Bimagrumab plus semaglutide enhances fat loss and muscle preservation. In: Regeneron. 2025. https://www.regeneron.com. Accessed 1 Nov 2025.

[35] Choi SJ, et al. The potential role of human endogenous retrovirus K in glioblastoma. J Clin Invest. 2024;(13):e12075688. 10.1172/JCI170885. 10.1172/JCI170885 PMC10313359 37395278

[36] Heymsfield SB, Aronne LJ, Montgomery P, et al. Bimagrumab plus semaglutide alone or in combination for the treatment of obesity: a randomized phase 2 trial. Nat Med. 2026;32:869–82. 10.1038/s41591-026-04204-0. 41772149 10.1038/s41591-026-04204-0 PMC13004672

[37] Dalton JT, et al. Enobosarm improves lean body mass and function. J Clin Endocrinol Metab. 2013;98:199–208. 10.1210/jc.2012-3692.23144469

[38] Dobs AS, Boccia RV, Croot CC, et al. Effects of enobosarm on muscle wasting and physical function in patients with cancer: a double-blind, randomised controlled phase 2 trial. Lancet Oncol. 2013;14:335–45. 10.1016/S1470-2045(13)70055-X. 23499390 10.1016/S1470-2045(13)70055-X PMC4898053

[39] Bedi H, Hammond C, Sanders D, et al. Drug-induced liver injury from enobosarm (ostarine), a selective androgen receptor modulator. ACG Case Rep J. 2021;8:e00518. 10.14309/crj.0000000000000518. 34368386 10.14309/crj.0000000000000518 PMC8337042

[40] Collet TH, Litherland J, et al. Intra-gastric balloon as an adjunct to lifestyle support in severely obese adolescents; impact on weight, physical activity, cardiorespiratory fitness and psychosocial well-being. Int J Obes (Lond). 2017;41(4):163–9. 10.1038/ijo.2016.192. 10.1038/ijo.2016.192 PMC5382282 27795553

[41] Simmler C, Pauli GF, Chen SN. Phytochemistry and properties of glabridin. Fitoterapia. 2013;90:160–84. 10.1016/j.fitote.2013.07.003. 23850540 10.1016/j.fitote.2013.07.003 PMC3795865

[42] Noureddin M, et al. TERN-501 monotherapy and combination therapy with TERN-101 in metabolic dysfunction-associated steatohepatitis: the randomized phase 2a DUET trial. Nat Med. 2025;31:2297–305. 10.1038/s41591-025-03722-7. 40500414 10.1038/s41591-025-03722-7 PMC12283411

